# Minor Physical Anomalies and Congenital Malformations Among Children with Psychotic Symptoms: An Exploratory Descriptive Study with Illustrative Clinical Cases

**DOI:** 10.3390/brainsci16060604

**Published:** 2026-06-01

**Authors:** Zuzanna Ewa Wiśniewska, Przemysław Temistokles Zakowicz, Maria Skibińska

**Affiliations:** 1 Department of Neural Engineering and Space Medicine, Collegium Medicum, University of Zielona Góra, 65-001 Zielona Góra, Poland; zuzzawisniewska@gmail.com (Z.E.W.); mariaski@ump.edu.pl (M.S.); 2 Centre for Child and Adolescent Treatment in Zabór, 66-003 Zabór, Poland; 3 Department of Psychiatric Genetics, Poznan University of Medical Sciences, 60-806 Poznań, Poland

**Keywords:** minor physical anomalies (MPAs), neurodevelopmental markers, dysmorphology, psychosis spectrum, very early-onset schizophrenia (VEOS)

## Abstract

**Highlights:**

**What are the main findings?**

- Among the 19 patients with primary psychotic symptoms, seven were diagnosed with very early-onset schizophrenia (VEOS).
- Minor physical anomalies (MPAs) were detected in 42% of participants; three patients were diagnosed with major congenital malformations: Arnold–Chiari malformation type I, incomplete hippocampal inversion, and temporal cortex malformation.

**What are the implications of the main findings?**

- Meticulous head-to-toe examination of pediatric psychotic patients is essential to ensure that minor physical anomalies are not overlooked.
- Minor physical anomalies (MPAs) are subtle phenotypic indicators of neurodevelopmental aberrations.

**Abstract:**

**Background/Objectives**: Minor physical anomalies (MPAs) are subtle morphological markers of disrupted neuroectodermal development occurring during early gestation. Their increased prevalence has been reported in several neurodevelopmental and psychiatric conditions, including schizophrenia. However, data on MPAs in pediatric psychosis remain limited. This exploratory descriptive study aimed to characterize the occurrence of MPAs and congenital malformations in children presenting with early-onset psychotic symptoms and to illustrate the clinical heterogeneity through two representative cases. **Methods**: All participants underwent a comprehensive head-to-toe examination by a trained child and adolescent psychiatrist to identify MPAs. Key anatomical regions, including the face, hair vortex, palate, and extremities, were systematically photographed. Results were corroborated by MRI brain screening and consulted with a clinical geneticist. Anomalies were classified using the standardized Elements of Morphology terminology. **Results**: Among the 19 study participants, seven (37%) were diagnosed with very early-onset schizophrenia (VEOS). Identified minor physical anomalies included epicanthus (*n* = 5), café au lait spots (*n* = 2), and discoloured spots (*n* = 1). Furthermore, major congenital malformations were detected, specifically Arnold–Chiari malformation type I (*n* = 1), incomplete hippocampal inversion (*n* = 1), and a temporal cortex malformation (*n* = 1). **Conclusions**: MPAs and selected neuroanatomical anomalies were observed in a subset of children with early-onset psychotic symptoms. While the small sample size and absence of a control group limit interpretability, these exploratory findings suggest that systematic physical examination may provide supportive clinical information in the assessment of pediatric psychosis. Larger, controlled studies are needed to clarify whether specific MPAs may serve as early markers of neurodevelopmental vulnerability.

## 1. Introduction

A thorough examination in pediatric practice seems to be crucial for correct diagnosis. A head-to-toe examination may reveal nonspecific physical abnormalities, raising questions about the developmental origin and potential impact on patients’ functioning.

While dysmorphic features may provide the initial clue prompting suspicion of a genetic disorder, especially in psychiatric practice, clinicians often encounter a wide range of minor physical anomalies with no apparent connection. Minor anomalies (MPAs) are defined as subtle signs of developmental abnormalities, mainly in the limbs, head and particular parts of the face such as the eyes, ears, mouth, nose, and tongue [1]. Congenital malformations are single or multiple defects in the morphogenesis of organs or body parts. They can be identified at birth or during fetal life. Congenital malformations are typically classified into major defects, causing serious health consequences, impairing bodily function and shortening life, and minor defects (minor physical anomalies), mainly of cosmetic importance. MPAs do not cause serious health consequences, but may be an indication for further investigation and analysis of the patient’s behaviour. Their presence is helpful in the diagnosis of other, larger morphogenic abnormalities, like in the case of neurofibromatosis [2]. MPAs are thought to arise from genetic or environmental factors, often interacting with one another. Compromised fetal well-being during pregnancy, stemming from factors such as toxin exposure, hypoxia, hemorrhage, or maternal infections, significantly impacts development. An association between MPAs and various neuropsychiatric disorders such as epilepsy, autism, attention deficit/hyperactivity disorder, intellectual disability, schizophrenia, and bipolar disorder has been reported [3]. Based on these findings, identifying MPAs might provide a helpful rationale for conducting further, more detailed patient examinations [4]. The presence of MPAs indicates an early disruption in the simultaneous development of the surface ectoderm and neuroectoderm, so it may be an indication of events occurring in the developing brain [5]. Most MPAs are thought to occur during the first or early second trimester of pregnancy. As this period is critical for central nervous system development, any defects arising during this stage are considered physical markers of intrauterine brain development [6]. We can benefit from information about neurodevelopment defects in mental illnesses thanks to modern neuroimaging techniques. Moreover, MPAs can provide information about a person’s susceptibility to a disorder, even before the first symptoms indicative of the disease appear [7]. Minor physical anomalies usually occur in complex anatomical areas such as the face or hands. As the largest and most superficial organ, the skin is uniquely accessible for clinical examination. Due to its shared ectodermal origin with the nervous system, it can reveal subtle markers of neurodevelopmental abnormalities [8]. Furthermore, MPAs frequently cluster within families affected by psychiatric disorders [9,10]. The high frequency of minor physical anomalies in schizophrenic patients suggests early neuroectodermal abnormalities. Many mental disorders have prepubertal origins. Although schizophrenia typically onsets after puberty, studies in individuals at high risk, along with longitudinal and retrospective studies, have shown that the fully developed disorder is often preceded by impairments that manifest earlier in development [11]. Among children with childhood-onset schizophrenia, 67% show premorbid disturbances in the social, motor, and language domains. These children often also present with learning disabilities and appear to have comorbid mood or anxiety disorders prior to the onset of psychotic symptoms [12]. Psychosis spectrum occurring in the developmental age suggests a prominent role of neurodevelopmental abnormalities. Current perspectives on schizophrenia emphasize its neurodevelopmental origin, highlighting various genetic and/or environmental risk factors that contribute to aberrant brain development during pregnancy and early childhood. The investigation of minor physical anomalies in psychosis dates back to the earliest research on schizophrenia, with the first descriptions provided by Kraepelin and Clouston [13].

Here we present an exploratory, descriptive study on minor physical anomalies in child psychiatry practice, including a presentation of two cases. The description was based on hospital care patients, assessed in 2020, admitted to the Centre for Child and Adolescent Treatment in Zabór, Zielona Góra, Poland (tertiary referral centre for western Poland). In the analysis we included all admitted patients before the 12th year of life exhibiting psychotic symptoms.

## 2. Materials and Methods

### 2.1. Subjects and Protocol

The study was approved by the local Bioethics Committee at Poznan University of Medical Sciences, Poland (reference number 1066/19). Inclusion in the study was preceded by an informed consent of the caregiver, due to local law regulations, and oral assent from the child. All studied patients were recruited at the Centre for Child and Adolescent Treatment in Zabór, Zielona Góra, Poland (a tertiary referral centre for western Poland). Inclusion criteria were the presence of primary psychotic symptoms, defined as positive symptoms: hallucinations, disorganized thinking, and grossly disorganized behaviour. The study group comprised 19 participants, including 4 females and 15 males. All patients included in the study were younger than 12 years. We excluded patients with diagnosed genetic disorders (like neurofibromatosis, *n* = 2, Smith–Lemli–Opitz Syndrome, *n* = 1, or pachygyria, *n* = 1).

### 2.2. Dysmorphological Examination

All study participants were thoroughly examined (head to toe) searching for minor physical anomalies by a trained child and adolescent psychiatrist. Following parts of the patients’ body were photographed routinely in an anonymized model: hands and palms, feet, face, hair vortex and palate. For each minor anomaly found in different regions of the body, separate photography was made. All of the patients were provided with an MRI central nervous system screening examination. Obtained material was consulted with the clinical geneticist, with expertise in dysmorphology, and the anomalies were classified according to the Elements of Morphology human malformation terminology.

### 2.3. Diagnostic Process and Differential Diagnosis

The diagnostic process followed a comprehensive approach to ensure clinical accuracy. Primary psychiatric diagnoses were established based on the criteria of the International Classification of Diseases (ICD-10). To perform a rigorous differential diagnosis and exclude potential secondary causes of psychosis, all patients underwent thorough neurological consultations.

## 3. Results

### 3.1. Clinical and Demographic Data

From the hospital admissions in 2020, 19 patients fulfilled the inclusion criteria. The mean age was 10 (±2) years, with a minimal age of 7. Out of 19 patients, seven (37%), were diagnosed with very early-onset schizophrenia (VEOS), following the ICD-10 criteria, and the rest of the study group (*n* = 12, non-VEOS) was diagnosed with acute and transient psychotic disorder (n-9) and schizotypal disorder (*n* = 3). Out of 19 patients, two were with genetic burden of schizophrenia and one with completed suicide in first-degree relatives. Detailed comparison of VEOS vs. non-VEOS patients regarding demographic and clinical data is presented in the table below (Table 1).

### 3.2. Minor Physical Anomalies (MPAs) and Congenital Malformations in the Study Group

In the study population we identified the following minor physical anomalies: epicanthus (*n* = 5), café au lait spots (*n* = 2), and discoloured spots (*n* = 1). Regarding the congenital malformations, we found Arnold–Chiari malformation type I (*n* = 1), incomplete inversion of the hippocampus (*n* = 1) and temporal cortex cortical malformation (*n* = 1). The summary of MPAs and MRI findings is presented in the tables below (Table 2 and Table 3).

### 3.3. Case 1 Description: Cortical Malformation and Primary Psychotic Disorder

A 12 year old boy was admitted to the psychiatric emergency department due to progressive social isolation and disturbed communication. In the caregivers’ relation, the child was for the last few weeks complaining about the loss of attention during classes. Most of the time at home, the patient was writing in a notebook illogical sentences in a disorganized manner (e.g., “they told that she is not normal”, “the girl is not doing well”). In psychiatric examination at the admission, no developmental disturbances were found; the pregnancy and early-developmental age were not affected by risk factors, as well as the genetic family background. The intellectual functioning of the patient was assessed previously as high with no disabilities in the learning process. The direct assessment of the psychiatric state revealed the presence of auditory vocal pseudo-hallucinations (localized in the patients’ head) described by the patient as “not known voices of different people”, with no affective component (persecutory/grandiosity/minority content). Admission diagnosis was made as paranoid schizophrenia following IDC-10 criteria (presence of auditory hallucinations). The child did not exhibit any signs or symptoms in the neurological examination; MR imaging was made revealing the cortical malformation in the temporal cortex of the dominant hemisphere (left). The pharmacological treatment was administered with SGA (aripiprazole) with successful clinical remission of symptoms.

### 3.4. Case 2 Description: Very Early-Onset Schizophrenia in Child with Complex Trauma 

An 8 year old boy was admitted to the psychiatric emergency department as the outcome of paramedics’ intervention in the institutionary care system: behavioural disturbances, aggression, and suicidal declarations. The child was placed in institutional care due to maltreatment in the family. The complete neurodevelopmental and pregnancy data were not achieved due to lack of compliance from the family system. The child was institutionalized in the 6th year of life and diagnosed with developmental speech delay with autism spectrum disorder and reactive attachment disorder. The admission psychiatric examination revealed visual and auditory hallucinations, bizarre delusions of influence (“a demon controls my thoughts”), autoaggressive behaviours such as self-strangulation, and persecutory ideas. At admission, verbal contact was difficult; he avoided eye contact. His mood was lowered and dysphoric and his affect was blunted. He reported perceptual disturbances of the visual modality (“I see all the terrible things, sparks, from these sparks monsters are formed”), while his statements about auditory hallucinations were ambivalent (“during the day I see and hear Chinese signs”). His thought content revealed psychiatric automatisms: delusions of influence and control (“I have a demon that roars inside me, he directs me, inserts thoughts, puts dreams into me”). Initially during hospitalization he presented paroxysmal autoaggressive behaviour, psychomotor agitation, and aggression directed at staff. The neurological and neuroimaging examination did not reveal significant data. The child was diagnosed with primary very-early onset schizophrenia and managed with SGA. After 6–8 weeks of treatment with risperidone the symptoms were still exuberant and combined treatment was decided upon. Final remission of symptoms was achieved in two combined SGAs, risperidone and olanzapine, after ten weeks of hospitalization.

These clinical case studies aim to visually illustrate the presentation of very early-onset schizophrenia (VEOS) in relation to minor physical anomalies. Ultimately, such clinical manifestations should prompt clinicians to conduct a thorough examination and search for both MPAs and cortical malformations.

## 4. Discussion

Here we presented the description of patients’ population with a very early onset of psychotic symptoms. An analysis of the data in Table 1 reveals a distinct difference between the study groups (VEOS and non-VEOS), indicating a strong association between VEOS status and the prevalence of minor anomalies (85% vs. 17%). Although this finding suggests a potentially significant correlation, it should be interpreted with caution. The primary limitation of this study is the small sample size of the groups. Consequently, these findings require verification in future studies involving a larger population.

From the analysis of the cases we may set the conclusion about a highly heterogenous clinical picture also regarding congenital malformations and minor physical anomalies. Presence of these morphological features indicates the need for thorough examination and differential diagnosis among the youngest psychiatric patients. The discussion session will provide a brief overview of the findings.

### 4.1. Café au Lait Spots

These are flat hyperpigmented lesions of light to dark brown colour. They are observed from birth in approximately 3% of all newborns and occur generally in at least 10% of the population. They may not be visible until infancy or early childhood in fair-skinned individuals. Their most common location is the trunk and limbs, but they can occur in any part of the body. They are characteristically present from birth, but can also appear early in life. They can increase in number and size with age. There are two types of cafe-au-lait stains: “coast of California” with regular and clearly demarcated margins, and “coast of Maine” which is less common and is usually larger and solitary [14].

Cafe-au-lait spots can accompany a number of genetic diseases, like NF-1, Legius syndrome (NFLS), familiar multiple CALMs syndrome, or McCune and Albright syndrome. As not obvious features, café au lait spots are met also in tuberous sclerosis (TSC-1 and TSC-2), Fragile X syndrome (FraX), or multiple endocrine neoplasia type 2 (MEN 2).

### 4.2. Discoloured Spots

Discoloured spots (macule hypochromicae), otherwise known as ash leaf spots, are oval or leaf-shaped whitish discolorations, usually visible on the back. They contain melanocytes without melanin. These lesions occur in around 90% of patients with tuberous sclerosis [15]. The ash leaf-shaped patches on Wood’s Lamp examination show enhanced hypopigmentation [16]. They are a very characteristic symptom of the disease. The presence of three or more discolouration spots is a diagnostic criterion for tuberous sclerosis. Tuberous sclerosis is an autosomal dominantly inherited disease. It is characterized by epileptic seizures and intellectual deficits, as well as skin lesions such as discolouration patches and facial hemangiomas. Tuberous sclerosis is associated with two genes: TSC1 on chromosome 9 and TSC2 on chromosome 16. Mutations in either of these genes result in inactivation of the mTOR kinase inhibitor complex, which disrupts cell division and promotes tumour formation [17]. People with TSC are more likely to develop some forms of psychopathology. The most common neuropsychiatric symptoms include autism spectrum disorder, intellectual disability, depressive and anxiety disorders, and cognitive dysfunction [18]. Intellectual disability of varying degrees in TSC affects approximately 40–60% of patients, with TSC2 more commonly affected. Autism spectrum disorders affect 40–50% of patients, and ADHD affects 30–50%. More than half of those with TSC exhibit behavioural difficulties. The most common of these include aggression, tantrums, self-injury, depressed mood, anxiety, restlessness, poor ability to concentrate, and hyperactivity. Children with TSC are also characterized by difficulties in learning, prolonged attention, spatial memory and performing several activities simultaneously [18,19].

### 4.3. Cortical Malformations

Malformations are primary developmental disorders that arise during the embryonic period.

Cortical malformations comprise a heterogeneous group of structural brain anomalies, associated with complex neurodevelopmental disorders and diverse genetic and non-genetic aetiologies [20]. Common clinical consequences of cortical malformations are epilepsy, developmental delay, intellectual disability, autism, and schizophrenia. These disorders may co-occur in the same individual, but they can also present independently. Proliferation, neuronal migration and postmigratory development are the three main stages of cortical development. The previously mentioned phases overlap at some points. Proliferation continues after the onset of neuronal migration, and postmigratory development begins before neuronal migration ends [21,22,23]. In the human brain, neuronal migration starts when the first neurons are formed, around the fifth week of pregnancy. It reaches a peak between the third and fifth month of pregnancy and ends around the 24th to 28th gestational week. These processes continue intensively until term and persist into the postnatal period [24,25]. The mechanism that causes malformations is not fully understood.

However, it can be hypothesized that some of these abnormalities are due to underlying genetic mutations that disrupt encoded proteins. De novo mutations, arising during gametogenesis or postzygotic development, are increasingly recognized as causes of epilepsy, intellectual disability, and autism spectrum disorders. Some malformations associated with postzygotic disorders cause somatic mosaicism. An example is tuberous sclerosis, in which point mutations of the TSC2 gene have been identified [26]. Sometimes the defect takes the form of a malformation syndrome, but it can also occur singly and be associated with a specific anatomical area. This is a group of disorders that often manifest as developmental delay, cerebral palsy or convulsion. Malformations that occur at a later stage of embryonic development are less complex and associated with milder clinical consequences than those occurring early in development.

### 4.4. Incomplete Inversion of the Hippocampus

The hippocampus serves a critical function in memory and cognition. It is located in the temporal lobe between the choroidal fissure and the parahippocampal gyrus [27]. Incomplete hippocampal inversion (IHI), also called hippocampal malrotation, is present in about 20% of healthy individuals. It is an atypical variant of the hippocampus [28]. The hippocampus develops between the 8th and 21st weeks of pregnancy. Hippocampal inversion occurs in fetal life. It is a process that involves the dentate gyrus and cornu ammonis extending around the hippocampal sulcus [29].

If this process fails, incomplete hippocampal inversion occurs, which is characterized by a rounded hippocampus along with a vertically oriented hippocampal sulcus [30]. The volume of the hippocampus increases rapidly until the age of two years and then slowly increases until the teenage years. IHI was initially identified in patients with malformations of cortical development and/or agenesis of the corpus callosum [31]. IHI has been observed with a higher frequency in people with a microdeletion of chromosome 22q11.2 and in epilepsy populations [31]. Scientific research has shown that IHI is a common anatomical pattern in healthy individuals, occurs much more frequently in the left hemisphere, and is associated with more widespread morphological changes outside the hippocampus [32].

### 4.5. Epicanthus

The epicanthal fold, or epicanthus, is defined as a distinctive cutaneous drape overlying the medial canthus. Although this anatomical feature represents a characteristic phenotypic trait in East Asian populations, it is fundamentally a ubiquitous finding during fetal development across all ethnic groups. Morphological expression of the fold varies significantly in degree and is typically attributed to localized vertical skin deficiency and heightened tension, often exacerbated by the bridging of underlying subcutaneous tissue [33].

### 4.6. Arnold–Chiari Malformation Type I

Chiari malformation type I (CM-I) is a complex disorder of the craniocervical junction, first characterized in 1891 by Austrian pathologist Hans Chiari in relation to cerebellar ectopy and hydrocephalus. Currently recognized as a spectrum of congenital anomalies, CM-I exhibits significant heterogeneity in etiology, age of onset, and clinical presentation, with an estimated incidence of approximately 1 in 1000 births [34]. Furthermore, the condition has been documented in association with various rare genetic syndromes and multisystem congenital disorders [35]. From a pathophysiological perspective, classical CM-I is primarily attributed to an underdeveloped occipital bone. This structural deficiency leads to a constricted posterior cranial fossa (PCF), the volume of which is insufficient to accommodate a morphologically normal cerebellum. This overcrowding hypothesis—rather than a primary neurodevelopmental defect of the cerebellum itself—is supported by surgical outcomes; following the expansion of the PCF through decompression, the cerebellar tonsils often ascend and regain a regular configuration. Early-onset schizophrenia (EOS) is a clinical entity characterized by a particularly severe course and prognosis. Although the prevalence of full-blown psychotic episodes in the developmental population remains low, their manifestation constitutes a highly traumatic event for the entire family system. Such a diagnosis necessitates the implementation of long-term therapeutic strategies and the management of challenges inherent to a chronic condition. In the aetiopathogenesis of the disorder, alongside genetic predispositions, environmental determinants play a pivotal role.

Furthermore, subtle morphological anomalies may provide supportive clinical information into an individual’s susceptibility to a given disorder, manifesting even prior to the emergence of prodromal symptoms. The skin represents the most accessible medium for clinical examination. Due to their shared ectodermal origins, the integumentary system and the central nervous system are intrinsically linked, rendering cutaneous markers potential indicators of early neurodevelopmental disruptions. Unfortunately, a limitation of this study is the lack of a healthy control group, which makes establishing a highly certain diagnosis and drawing a definitive association difficult.

## 5. Conclusions

Contemporary clinical practice is evolving towards a paradigm dominated by advanced laboratory and imaging diagnostics, which frequently results in the marginalization of direct physical examination. While the dynamic technological progress in medicine is undeniably beneficial, it remains crucial to maintain rigour regarding fundamental diagnostic procedures, such as palpation and observation.

Specific clinical vigilance and perceptiveness in the phenotypic assessment of a patient can be of significant importance to both the scientific research process and the optimization of the patient’s developmental trajectory. Identifying correlations between specific morphological anomalies and genetic conditions could represent a helpful step toward future diagnostic improvements. Our study is not without its constraints. The most significant limitation lies in the restricted sample size of patients suffering from schizophrenia. Given that certain anomalies—such as epicanthic folds and café au lait spots—were observed in several patients, further longitudinal study is warranted. Examining a larger cohort over an extended period would facilitate more definitive findings. In conclusion, while technology serves as a powerful ally, a thorough physical examination and an insightful clinical history remain the cornerstones of diagnosis. Meticulous observation and documentation of minor physical anomalies may provide the essential evidence required to discern recurring patterns among children with schizophrenia. Much as the epicanthic fold has become a hallmark of Trisomy 21, sustained clinical observation of these subtle markers may ultimately enable us to determine whether a distinct physical profile exists for neurodevelopmental pathways within psychiatry.

## Figures and Tables

**Table 1:** Comparison of VEOS vs. non-VEOS group regarding demography and clinical data.

	VEOS	Non-VEOS
*n*	7	12
Mean age ±SD	11 ± 2	9 ± 1
Sex	M = 5, F = 2	M = 10, F = 2
Family burden of schizophrenia	1	1
Minor physical anomalies (*n*,%)	6 (85%)	2 (17%)

**Table 2:** Summary of minor physical anomalies and MRI findings in studied population.

Feature/Anomaly	*n*	% of Patients
Minor physical anomalies	8	42%
Epicanthus	5	26%
Café au lait spots	2	10%
Discoloured spots (ash-leaf)	1	5%
MRI anomalies	3	16%
Arnold–Chiari I	1	5%
Incomplete hippocampal inversion	1	5%
Temporal cortex malformation	1	5%

**Table 3:** Detailed case series description.

Case ID	Sex	Age	Diagnosis (ICD-10 Code)	MPA (Yes/No)	Type of MPA	MRI Findings	Family Burden of Schizophrenia
BD_1	M	8	F20	No			
BD_2	M	12	F20	Yes	Epicanthus	Arnold–Chiari t.I	
BD_3	M	9	F23	No			
BD_4	F	9	F20	No			
BD_5	M	11	F23	Yes	Café au lait spots		
BD_6	M	8	F23	No			
BD_7	M	9	F23	No			
BD_8	M	10	F23	No			Yes
BD_9	F	10	F23	No			
BD_10	M	11	F20	No			
BD_11	M	12	F20	Yes	Epicanthus	Cortical dysplasia	
BD_12	M	12	F20	Yes	Epicanthus	Hippocampal inversion	
BD_13	F	12	F20	Yes	Epicanthus, wide nasal bridge		Yes
BD_14	M	9	F23	No			
BD_15	M	10	F23	Yes	Café au lait spots		
BD_16	M	10	F23	Yes	Discoloured spots		
BD_17	M	9	F21	Yes	Epicanthus		
BD_18	F	10	F23	No			
BD_19	M	7	F23	No			

## Data Availability

The raw data supporting the conclusions of this article will be made available by the authors on request due to privacy.

## Abbreviations

The following abbreviations are used in this manuscript:

MPAs	Minor physical anomalies
VEOS	Very early-onset schizophrenia
MRI	Magnetic resonance imaging
SGA	Second-generation antipsychotic
CALM	Café au lait macule
NF-1	Neurofibromatosis type 1
MEN 2	Multiple endocrine neoplasia type 2
FraX	Fragile X syndrome
IHI	Incomplete hippocampal inversion
ID	Intellectual disability
CM-1	Chiari malformation type I
PCF	Posterior cranial fossa
EOS	Early-onset schizophrenia
ADHD	Attention deficit/hyperactivity disorder
NFLS	Legius syndrome
TSC-1/TSC-2	Tuberous sclerosis complex genes
TSC	Tuberous sclerosis complex
